# Blood Pressure and Tooth Loss: A Large Cross-Sectional Study with Age Mediation Analysis

**DOI:** 10.3390/ijerph18010285

**Published:** 2021-01-02

**Authors:** José João Mendes, João Viana, Filipe Cruz, Dinis Pereira, Sílvia Ferreira, Paula Pereira, Luís Proença, Vanessa Machado, João Botelho, João Rua, Ana Sintra Delgado

**Affiliations:** 1 Clinical Research Unit (CRU), Centro de Investigação Interdisciplinar Egas Moniz (CiiEM), Egas Moniz—Cooperativa de Ensino Superior, 2829-511 Caparica, Portugal; jmendes@egasmoniz.edu.pt (J.J.M.); jpm.viana.1@gmail.com (J.V.); cacelafilipe@gmail.com (F.C.); adtper@gmail.com (D.P.); vmachado@egasmoniz.edu.pt (V.M.); jrua@egasmoniz.edu.pt (J.R.); anasintradelgado@egasmoniz.edu.pt (A.S.D.); 2 Evidenced-Based Hub, CiiEM, Egas Moniz—Cooperativa de Ensino Superior, 2829-511 Caparica, Portugal; lproenca@egasmoniz.edu.pt; 3 Patologia Clínica, Centro Hospitalar Lisboa Ocidental, 1449-005 Lisboa, Portugal; silviadfsilva@gmail.com; 4 Grupo de Estudos em Nutrição Aplicada (GENA), CiiEM, Egas Moniz—Cooperativa de Ensino Superior, 2829-511 Caparica, Portugal; pereira.paula1@gmail.com; 5 Quantitative Methods for Health Research (MQIS), CiiEM, Egas Moniz—Cooperativa de Ensino Superior, 2829-511 Caparica, Portugal

**Keywords:** hypertension, high blood pressure, oral health, dental medicine, public health, Edentulism, tooth loss, age, mediation analysis

## Abstract

We aimed to investigate the association between blood pressure (BP) and tooth loss and the mediation effect of age. A cross-sectional study from a reference dental hospital was conducted from September 2017 to July 2020. Single measures of BP were taken via an automated sphygmomanometer device. Tooth loss was assessed through oral examination and confirmed radiographically. Severe tooth loss was defined as 10 or more teeth lost. Additional study covariates were collected via sociodemographic and medical questionnaires. A total of 10,576 patients were included. Hypertension was more prevalent in severe tooth loss patients than nonsevere tooth lost (56.1% vs. 39.3%, *p* < 0.001). The frequency of likely undiagnosed hypertension was 43.4%. The adjusted logistic model for sex, smoking habits and body mass index confirmed the association between continuous measures of high BP and continuous measures of tooth loss (odds ratio (OR) = 1.05, 95% CI: 1.03–1.06, *p* < 0.001). Age mediated 80.0% and 87.5% of the association between periodontitis with both systolic BP (*p* < 0.001) and diastolic BP (*p* < 0.001), respectively. Therefore, hypertension and tooth loss are associated, with a consistent mediation effect of age. Frequency of undiagnosed hypertension was elevated. Age, gender, active smoking, and BMI were independently associated with raised BP.

## 1. Introduction

Hypertension is estimated to affect 1.56 billion (1.54–1.58) people in 2025 [1] and is defined as a persistent high systemic arterial blood pressure (BP) [2]. This raised BP is dependent on several risk factors, such as age, sex, obesity, excessive alcohol consumption, smoking, high dietary salt intake, physical inactivity and stress [3]. Furthermore, this condition is a primary modifiable risk factor for a number of illnesses, for instance, cardiovascular or cerebrovascular diseases [4]. Hypertensive populations have a greater probability of precocious perish [5]. In addition, uncontrolled hypertension may also precipitate stroke events and cognitive impairment, and it contributes to increased mortality rate [6]. Recently, high BP was associated with oral conditions, mainly tooth loss, caries and periodontal disease [7,8,9,10,11,12].

Tooth loss may be seen as the worst-case scenario in oral health. In 2015, 4.1% of the world population (276 million) was reported to be edentulous [13]. Overall, the primary causes of tooth loss are dental caries and severe forms of periodontal disease [14]. Tooth loss is one of the leading oral conditions causing disability-adjusted life years (DALY) in almost all global regions [13], and might have a direct implication on patients’ psychological health [15], aesthetics [15] or dietary/nutrition state [16,17]. Moreover, the increase in missing teeth might have an impact on patients’ oral health-related quality of life [18].

Several lines of evidence have addressed the association between tooth loss and high BP [4,8,9,12]. In a French cohort of people under 65 years old, hypertension was associated with higher levels of missing teeth, gum bleeding, masticatory dysfunction and dental plaque [19]. Furthermore, abnormal elevated systolic BP (SBP) levels were correlated with tooth loss in adults <60 years in a population-based study in Brazil [4]. Considering the interplay between hypertension and tooth loss, dental care appointments constitute an environment where BP is commonly measured and, therefore, might play a noteworthy responsibility in forwarding suspected undiagnosed hypertensive patients to seek proper care [8]. Exploring the association of both conditions and the levels of undiagnosed hypertensive patients in dental units would be of great public health interest.

With the present study, we intend to evaluate a relationship between measurements of BP and tooth loss and the mediation effect of age, on a large cross-sectional study from a national reference dental care clinic. As a secondary aim, we investigated the prevalence of potential undiagnosed hypertension patients.

## 2. Materials and Methods

Ethics approval for this research was granted by the Egas Moniz Ethics Committee (Ref: 733) and under the Declaration of Helsinki, as revised in 2013. This primary cross-sectional study was performed following the Strengthening the Reporting of Observational Studies in Epidemiology (STROBE) guidelines [20] (Supplementary Materials Table S1).

### 2.1. Setting

This study uses data from the Egas Moniz Dental Clinic (EMDC) database, a university clinic, located in the southern Lisbon Metropolitan Area, that provides dental health services to the general public [21]. This cross-sectional study was an uninterrupted data analysis of the triage dental appointments database. In the EMDC, a triage appointment is a mandatory clinical step for every patient to appropriately diagnose and plan the oral status of the patient. It has collected data from September 2017 until July 2020. 

### 2.2. Participants

To be eligible to participate in the present study, the following inclusion criteria were determined: willing to participate and have signed informed consent; had a triage appointment; had a panoramic radiograph and had carried out blood pressure measurement. The exclusion criteria were as follows: being less than 18 years old and, for women, being pregnant, considering the risk of existing gestational hypertension in this case [22]. 

### 2.3. Variables

Using an automated sphygmomanometer device (CardioAfib, Pic Solution^®^, Grandate, Italy), BP readings were carried out as a one-single measure [23]. Patients avoided caffeine, exercise and smoking in the 30 min before BP measurement. Moreover, patients remained seated for 3–5 min without talking or moving around before recording the BP reading, and patients were relaxed, sitting in a chair with their feet flat on the floor and their back supported. Both the patient and the observers did not talk during the test and measurement periods. The patient’s arm was resting on a desk, and the middle of the cuff was positioned on the patient’s upper arm at the level of the right atrium, with the bladder encircling 75–100% of the arm. SBP and diastolic BP (DBP) were recorded to the nearest value, and these readings were provided, both verbally and in writing, to each patient [24]. Overall average SBP, DBP and pulse were used in a continuous format. Further, hypertension was defined as values of SBP ≥ 140 mmHg or DBP ≥ 90 mmHg, or, the use of antihypertensive medication [25,26]. Arterial hypertension was categorized according to the 2018 European Society of Cardiology/European Society of Hypertension classification of office BP and definitions of hypertension grade [27].

Tooth loss assessment was performed based on oral examination and confirmation with a panoramic X-ray. Panoramic radiographs were made via digital Orthophos XG 5 DS/Ceph (Sirona Dental System, New York, NY, USA) at the Radiology Department of EMDC. Participants were categorized for tooth loss severity based on how many teeth they had lost: 10 or more—severe; lower than 10—nonsevere [11,12,28].

Additional study covariates were collected via sociodemographic and medical questionnaires. Among these covariates were gender, age and active smoking (currently smoking). The number of self-reported medical conditions was calculated as numerical (including diabetes mellitus (DM) that was confirmed using WHO criteria [29]). Among the possible medical conditions were asthma, congestive heart failure, coronary heart disease, angina, stroke, heart attack, emphysema, overweight, bronchitis, liver conditions, thyroid conditions and cancer. Measurements of height and weight were taken at the clinical exam and, body mass index (BMI) was calculated as kg/m^2^.

### 2.4. Statistical Analysis

To proceed with the analysis of the data, we used IBM SPSS Statistics v. 25.0 (IBM Corporation, Armonk, NY, USA). Continuous variables were represented in the form of mean and standard deviation (SD), and categorical variables were represented in the form of percentage (%) and frequency/cases (*n*). After validation of data normality and homoscedasticity, we employed the *t*-Student test to compare mean values according to the severity of tooth loss. Additionally, the chi-square test was used for comparison of categorical variables. To illustrate the relationship between people suffering from hypertension with tooth loss, we created a graph using scatter plots from the “ggplot2” package for R v. 4.0; to calculate the trend, we used “geom_smooth”. Multivariate logistic regression analyses were used to model the influence of potential factors in the relationship between tooth loss and hypertension. Odds ratio (OR) and 95% confidence intervals (95% CI) were calculated within the logistic regression analyses; for different adjustment levels, by using multiple linear regression analyses, we investigated the possible linear relationship between SBP and DBP and age, BMI and missing teeth. The model adjustment was made progressively by including sex, smoking habits, BMI (continuous measure) and age (continuous measure), respectively.

Finally, a mediation analysis was carried out to examine the mediating effect of age in the association of missing teeth with SBP and DBP. We defined three pathways in the mediation analysis: (1) exposure to mediator, (2) mediator to outcome (direct effect), and (3) exposure to outcome (total effect). The total effect was obtained through the sum of a direct effect and a mediated (indirect) effect. Percentage of the mediated effect was calculated using the formula: (mediated effect/total effect) × 100. A significance level of 5% was set in all inferential analyses.

## 3. Results

### 3.1. Baseline Characteristics 

From an initial sample of 11,021 participants, 445 were excluded after applying the exclusion criteria (413 patients less than 18 years old, 29 pregnant women and four patients who refused to take BP measurement). The final sample consisted of 10,576 patients, with an average of 44.9 (17.9) years of age and being predominantly female (59.7%) (Table 1). The average BMI for the sample was 25.5 (4.7) kg/m^2^, and 25.7% (*n* = 2722) were active smokers. Concerning BP, mean SBP and DBP were 135.3 (20.1) and 83.0 (12.3) mmHg, respectively. This sample presented an average of 5.8 (7.2) missing teeth. In addition, patients had at least one self-reported medical condition. Furthermore, 18.1% (*n* = 1919) reported to have hypertension and 21.1% (*n* = 2230) to use antihypertensive drugs. Overall, 43.2% (*n* = 5470) were classified as hypertensive according to BP measurements, and 43.4% (*n* = 3504) were categorized as hypertensive without any previous medical history of the disease or medication.

Specifically, participants with severe tooth loss were older (61.4 ± 12.4 vs. 39.9 ± 16.3, *p* < 0.001) and had higher BMI (25.5 (4.7) vs. 25.0 (4.5), *p* <0.001), SBP (142.1 (21.0) vs. 133.2 (19.3), *p* < 0.001) and DBP levels (83.5 (12.7) vs. 82.8 (12.2), *p* = 0.008). Furthermore, self-reported hypertension (22.2% vs. 4.8%, *p* < 0.001) and usage of antihypertensive drugs (21.7% vs. 19.2%, *p* = 0.008) were more prevalent in patients with severe tooth loss. The prevalence of hypertension at examination was found higher in severe tooth loss patients (56.1% vs. 39.3%, *p* < 0.001). Of those, 34.0%, 15.6% and 6.6% were diagnosed with grade 1, 2 and 3, respectively. In contrast, cases of optimal and normal blood pressure were more prevalent in patients with less than 10 teeth lost (10.7% and 12.8% vs. 18.3% and 20.7%, respectively).

**Figure 1:**
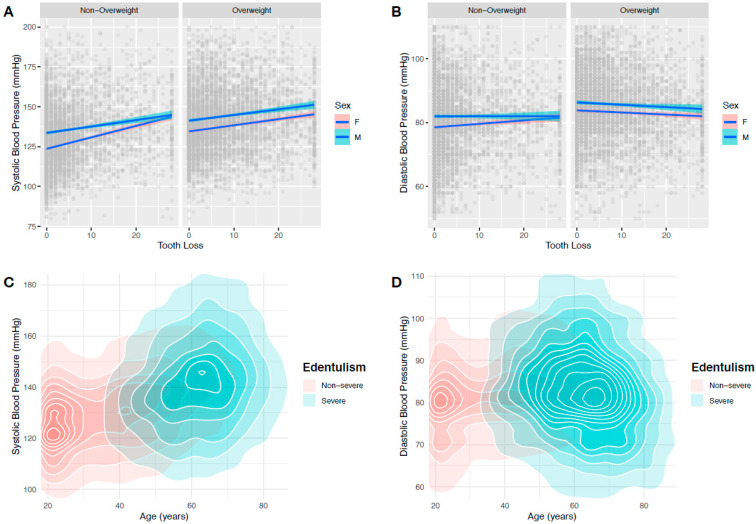
Scatter and contours plots showing the effect of different variables (age, weight status, sex and tooth loss severity) in the components of blood pressure. The increase in tooth loss was associated with (**A**) an increment in SBP in both nonoverweight and overweight participants, but (**B**) not so marked in DBP. In addition, a cluster of individuals with severe tooth loss is noted among older adults at higher levels of (**C**) SBP and (**D**) DBP.

Furthermore, we graphically explored the intricate association of age, sex, weight status and tooth loss with SBP and DBP (Figure 1). We confirmed a consistent association of SBP with tooth loss in both nonoverweight and overweight participants, though for DBP, this association was erratic (Figure 1A,B). Additionally, older adults were more associated with severe tooth loss and higher measures of SBP and DBP (Figure 1C,D).

### 3.2. Relationship between Hypertension and Tooth Loss

For the overall sample, when considering a crude model (Model 1) the presence of hypertension (odds ratio (OR) = 1.05, 95% CI: 1.05–1.06) and grade 3 hypertension (OR = 1.04, 95% CI: 1.03–1.05) were associated with the number of teeth lost (Table 2). This behaviour was maintained even when adjusting the model for sex, smoking habits and BMI (Table 2). In patients not taking antihypertensive medication, the risk of hypertension was similarly dependent on the increasing number of missing teeth (Model 1: OR = 1.06, 95% CI: 1.05–1.07) as well as for grade 3 (Model 1: OR = 1.05, 95% CI: 1.03–1.06). When adjusting for sex, smoking habits and BMI (Model 4), the same pattern of the global sample towards hypertension was observed (OR = 1.05, 95% CI: 1.03–1.06).

Then, multiple linear regression analyses investigated the linear relationship between SBP and DBP with age, BMI and missing teeth, according to the use of antihypertensive medications both for the overall sample (Table 3) and in a sex-specific analysis (Table 4). 

In the global sample, the obtained linear regression models confirmed that age and BMI were significantly related to SBP (β = 0.28, *p* < 0.001 and β =0.91, *p* < 0.001, respectively) and BMI with DBP (β = 0.59, *p* < 0.001). For patients reporting not using antihypertensive medications, a significant linear relationship of age, BMI and missing teeth with SBP was observed (β = 0.27, 0.93 and 0.07, respectively); however, for DBP, this relationship was only confirmed for BMI (β = 0.61, *p* < 0.001). Finally, for patients reporting antihypertensive use, both SBP and DBP were found to be significantly linear related to age and BMI.

### 3.3. Mediation Analysis of Age

There was evidence that the association between tooth loss and blood pressure (SBP and DBP) was mediated by age (Table 5). A significant model was obtained when age was included as a mediator of the association between missing teeth and SBP (missing teeth → age, *p* < 0.001, 95% CI: 1.21–1.28; age → SBP, *p* < 0.001, 95% CI: 0.34–0.39; and missing teeth → SBP, *p* < 0.73, 95% CI: 0.00–0.10). Age mediated 80% the link between missing teeth with SBP (β = 0.45, 95% CI: 0.42–0.49). Similarly, a significant model was obtained when age was included as mediation in the association between missing teeth and DBP (missing teeth → age, *p* < 0.001, 95% CI: 1.21–1.28; age → DBP, *p* < 0.001, 95% CI: 0.04–0.07; and missing teeth → DBP, *p* = 0.726, 95% CI: −0.03–0.04), with a significant mediation effect of 87.5% (β = 0.07, 95% CI: 0.05–0.09).

## 4. Discussion

With the present study, we investigated whether the components of BP were associated with the number of missing teeth. Our results demonstrate an association between office BP and tooth loss. Furthermore, the prevalence of likely undiagnosed hypertension patients was 43.4%. Then, we confirmed that these associations were significantly mediated by age as previously confirmed in other fields of research [8].

These results may have important implications: (1) our analyses are based on a consecutive large dataset from a dental clinic and may serve as reference information for future research; (2) the increase in BP may be associated with the number of teeth lost but is consistently age dependent; (3) the high prevalence of probable undiagnosed patients, detected in this national reference dental clinic, suggests a possible role of oral care providers in the surveillance of patients with apparent hypertensive levels and to refer them to seek proper care; (4) the average SBP of this population was high, which might be explained by the elevated average BMI observed.

Despite the existence of an association, DBP did not exhibit a relationship with tooth loss. It seems implausible that the existence of a direct biological mechanism could explain the difference in the association between SBP and DBP. Nevertheless, hypertension is seen as a result of high values of SBP and/or DBP, though in this specific sample, the association between hypertension and tooth loss was seen only via SBP values. Interestingly, both components are strongly age related within the association with missing teeth; however, DBP demonstrated high association with BMI, as shown previously [30,31,32,33]. 

The role of tooth loss as a possible risk factor for hypertension may certainly be the result of an indirect implication of oral health, rather than a direct influence, which is highly remote from a physiological point of view. Oral diseases such as caries and periodontal disease may participate in this equation, as they are the main responsible for tooth loss [34]. 

On the one hand, the mechanisms by which periodontitis is a possible risk factor for hypertension were further debated [7]. The main cause of periodontitis development is the accumulation of plaque around the tissues surrounding teeth [35]. These will be responsible for the development of a local inflammation which may develop into a systemic inflamed status that can result in vascular/perivascular alterations [36]. The latter can lead to vascular dysfunction which in turn may cause hypertension [37]. 

On the other hand, the association between caries and hypertension lacks biological foundation [38] and may be explained by the consequences in the individual’s diet and nutrition caused by tooth loss. People partially or totally toothless will ingest foods that have a more favourable consistency for their consumption [16]. Consequently, these impaired diets due to missing teeth are not the most suitable since they are nutritionally poor [39]. These impoverished food choices, along with poor physical activity, may lead to an increase in BMI and body fat. In turn, the increase in body fat might have systemic and cardiovascular repercussions, and may result in an increased BP [40]. Additionally, a high-intake sugar diet will likely increase the risk towards dental caries, one of the main causes of tooth loss [41]. However, a high-intake sugar diet has been shown to be associated with high BP, though there is still some uncertainty [42,43,44,45].

For these reasons, future studies should gather a detailed history of how the patient lost their teeth, with blood sample testing to ascertain systemic values and to obtain a more detailed report of the individual’s diet. 

There is already evidence that demonstrates a relationship between hypertension and tooth loss. Hitherto, the vast majority did not employ accurate methods to measure tooth loss, for instance, self-reported measures [9,11,12,28]. Thus, in our study, to be as precise as possible, we resorted to clinical confirmation combined with a thorough analysis via panoramic X-ray taken at our dental hospital. Another point to highlight is the large sample included, making these results highly compelling and, possibly, generalizable.

Furthermore, the effects of diabetes and smoking are important to consider in the hypertension/tooth loss association. First, hypertension is consistently linked with diabetes [46] and smoking habits [47]. In the same fashion, active smoking is a strong factor contributing to tooth loss [48] as well as uncontrolled diabetes [49], particularly in severe cases of periodontal destruction. In this sense, our results are in agreement with the literature, as we confirmed active smoking and diabetes as important confounding variables in this association; in other words, if present, they will contribute to higher odds of hypertension and severe tooth loss.

The frequency of undiagnosed hypertensive patients has been reported to range between 15%–50% [8,50,51,52,53,54]. Up to a certain level, our study shows a disturbing high count of unidentified high BP cases, and it was much more frequent in individuals with severe tooth loss. Despite the lack of consistent evidence, undiagnosed high BP may precipitate higher rates of cardiovascular mortality and morbidity [55,56,57,58]. As in Machado et al. [8], these results validate dental units as potential primary care locations for detecting cases of undiagnosed hypertension and, possibly, expanding to other clinical situations (such as diabetes). According to the recent Lancet Commission on hypertension, one of the pillars of prevention is universal access to measurement of BP through inexpensive BP monitors, and here, dental units may be highly helpful and resourceful [59]. Future longitudinal studies should investigate the accuracy and effectiveness of dental units in this prospective preventive role. 

### Strengths and Limitations

Although our investigation presents some visible strengths, it also has particular limitations. As this report is based on a cross-sectional survey, we cannot make any conclusions regarding a temporal connection or the deduction of causality between hypertension and missing teeth. Furthermore, the nonspecification of ex-smokers and the number of cigarettes in active smokers may be viewed as a limitation to bear in mind in future studies. Another limitation is the fact that our analysis builds on one BP measure, with inherent reported bias limiting the validity of these results [24]. However, this method resembles a more practical and usable approach to identify potential hypertensive patients within a crowded dental setting, and our results are in line with recent a national epidemiologic study [60]. Additionally, we were not able to introduce into the analysis confounders such as the history of the utilization of dental services, education background or family income, as they were not gathered during this period of time. Moreover, the periodontal status was not considered as it is not a standard diagnostic procedure of this triage appointment because the Periodontal Screening and Recording Index [61,62] is employed, and this shall be included in future studies since previous literature reported such association [8].

Another limitation is the patients’ inability to report, precisely, the cause of tooth loss. Recent studies in this regional area, but also at a national level, have demonstrated the serious ignorance regarding oral health, its consequences and oral hygiene habits [63,64,65,66,67,68]. Thus, it would be very risky to consider self-reported data regarding the cause of tooth loss. 

Further strengths regarding this investigation include a large number of participants, the comprehensive collection regarding medical history and the systematic clinical confirmation of missing teeth through clinical and radiograph procedures. Furthermore, this study assessed the mediation effect of age on this association, which can be seen as a novel result within the available literature. 

## 5. Conclusions

According to our data, office-measured BP and tooth loss are related, and age is a key mediator in this association. The prevalence of potential undiagnosed hypertensive patients was high, indicating the role of dental hospitals in the detection and referral to proper care. Age, gender, active smoking, and BMI were independently associated with raised BP. These results suggest that BP negatively impacts tooth loss rate.

## Figures and Tables

**Table 1:** Baseline characteristics of participants according to tooth loss severity.

Variable	Global (*n* = 10,576)	≥10 Teeth Lost (*n* = 2465)	<10 Teeth Lost (*n* = 8111)	*p*-Value *
Age, mean (SD) (years)	44.9 (17.9)	61.4 (12.4)	39.9 (16.3)	**<0.001**
Female gender, % (n)	59.7 (6312)	59.6 (1470)	59.7 (4842)	0.996
BMI, mean (SD) (kg/m2)	25.5 (4.7)	27.1 (4.7)	25.0 (4.5)	**<0.001**
Current smoker, % (n)	25.7 (2722)	27.0 (667)	25.3 (2055)	**<0.001**
SBP, mean (SD) (mmHg)	135.3 (20.1)	142.1 (21.0)	133.2 (19.3)	**<0.001**
DBP, mean (SD) (mmHg)	83.0 (12.3)	83.5 (12.7)	82.8 (12.2)	**0.008**
PP, mean (SD) (mmHg)	76.7 (2.8)	76.4 (2.9)	76.8 (2.7)	**<0.001**
Missing teeth, mean (SD)	5.8 (7.2)	17.1 (5.7)	2.4 (2.7)	**<0.001**
Medical conditions, mean (SD)	1 (1)	2 (1)	1 (1)	**<0.001**
Self-reported hypertension, % (n)	18.1 (1919)	22.2 (1800)	4.8 (119)	**<0.001**
Use of antihypertensive drugs, % (n)	21.1 (2230)	21.7 (1757)	19.2 (473)	**0.008**
Hypertension, % (n)	43.2 (5470)	56.1 (1384)	39.3 (3186)	**<0.001**
Blood Pressure, % (n)				
Optimal	16.5 (1747)	10.7 (264)	18.3 (1483)	**<0.001**
Normal	18.9 (1996)	12.8 (316)	20.7 (1680)	
High Normal	21.4 (2263)	20.3 (501)	21.7 (1762)	
Grade 1	27.8 (2943)	34.0 (838)	26.0 (2105)	
Grade 2	10.8 (1139)	15.6 (384)	9.3 (755)	
Grade 3	4.6 (488)	6.6 (162)	4.0 (326)	
Hypertension (excluding self-reported hypertension and taking antihypertensive drugs) (*n* = 8069), % (*n*)	43.4 (3504)	55.8 (1086)	39.5 (2418)	**<0.001**

BMI—body mass index; SBP—systolic blood pressure; DBP—diastolic blood pressure; PP—pulse pressure; * *t*-test for continuous variables, chi-square test for categorical variables, *p* < 0.05 denoted in bold.

**Table 2:** Odds ratios (OR) and correspondent 95% confidence intervals (95% CI) of risk towards hypertension and grade 3 according to the number of missing teeth, calculated within binary logistic regression analyses for different adjustment levels.

Variable	Hypertension	Hypertension Grade 3
All participants		
Model 1	1.05 (1.05–1.06) ***	1.04 (1.03–1.05) ***
Model 2	1.05 (1.05–1.06) ***	1.04 (1.03–1.05) ***
Model 3	1.05 (1.05–1.06) ***	1.04 (1.03–1.05) ***
Model 4	1.04 (1.03–1.05) ***	1.03 (1.02–1.05) ***
Model 5	1.00 (1.00–1.01)	1.01 (1.00–1.03)
Participants not taking antihypertensive medication (*n* = 8346)		
Model 1	1.06 (1.05–1.07) ***	1.05 (1.03–1.06) ***
Model 2	1.06 (1.05–1.08) ***	1.03 (1.01–1.06) *
Model 3	1.06 (1.05–1.08) ***	1.03 (1.01–1.06) *
Model 4	1.05 (1.03–1.06) ***	1.02 (1.00–1.05)
Model 5	1.00 (0.99–1.02)	1.00 (0.97–1.03)

Number of missing teeth was accounted as a continuous variable. OR is scaled per one missing tooth. Model 1—unadjusted model; Model 2—includes adjustment for sex; Model 3—includes adjustment for sex and smoking habits; Model 4—includes adjustment for sex, smoking habits and BMI; Model 5—includes adjustment for sex, smoking habits, BMI and age; * *p* < 0.05; *** *p* < 0.001.

**Table 3:** Multiple linear regression models for all participants and according to the use of antihypertensive medication for systolic blood pressure (SBP) and diastolic blood pressure (DBP).

Variable	Overall		No Antihypertensive Use (*n* = 8346)		Antihypertensive Use (*n* = 2230)	
	β Coefficient (SE)	*p*-Value	β Coefficient (SE)	*p*-Value	β Coefficient (SE)	*p*-Value
SBP (mmHg)						
Age (years)	**0.28 (0.01)**	**<0.001**	**0.27 (0.02)**	**<0.001**	**0.30 (0.03)**	**<0.001**
BMI (kg/m^2^)	**0.91 (0.04)**	**<0.001**	**0.93 (0.05)**	**<0.001**	**0.54 (0.06)**	**<0.001**
Missing teeth (n)	0.06 (0.03)	0.076	**0.07 (0.04)**	**0.049**	0.00 (0.07)	0.955
DBP (mmHg)						
Age (years)	0.01 (0.01)	0.135	0.01 (0.01)	0.511	**0.04 (0.06)**	**0.042**
BMI (kg/m^2^)	**0.59 (0.03)**	**<0.001**	**0.61 (0.03)**	**<0.001**	**0.54 (0.06)**	**<0.001**
Missing teeth (n)	−0.03 (0.02)	0.115	−0.02 (0.02)	0.415	−0.09 (0.05)	0.059

*p* < 0.05 denoted in bold.

**Table 4:** Multiple linear regression models according to sex and to the use of antihypertensive medication for systolic blood pressure (SBP) and diastolic blood pressure (DBP).

Variable	Females (Overall) (*n* = 6312)		Females not Taking Antihypertensive Use (*n* = 4937)		Females Taking Antihypertensive Use (*n* = 1375)		Males (Overall) (*n* = 4264)		Males not Taking Antihypertensive Use (*n* = 3409)		Males Taking Antihypertensive Use (*n* = 855)	
	β Coefficient (SE)	*p*-Value	β Coefficient (SE)	*p*-Value	β Coefficient (SE)	*p*-Value	β Coefficient (SE)	*p*-Value	β Coefficient (SE)	*p*-Value	β Coefficient (SE)	*p*-Value
SBP (mmHg)												
Age (years)	**0.29 (0.02)**	**<0.001**	**0.28 (0.02)**	**<0.001**	**0.32 (0.04)**	**<0.001**	**0.24 (0.02)**	**<0.001**	**0.24 (0.02)**	**<0.001**	**0.24 (0.05)**	**<0.001**
BMI (kg/m2)	**0.90 (0.05)**	**<0.001**	**0.914 (0.05)**	**<0.001**	**0.82 (0.11)**	**<0.001**	**0.74 (0.07)**	**<0.001**	**0.76 (0.08)**	**<0.001**	**0.70 (0.15)**	**<0.001**
Missing teeth (n)	0.07 (0.04)	0.065	0.08 (0.04)	0.065	0.04 (0.09)	0.690	0.08 (0.05)	0.114	0.10 (0.06)	0.087	0.02 (0.11)	0.896
DBP (mmHg)												
Age (years)	0.01 (0.01)	0.402	0.00 (0.01)	0.929	0.04 (0.02)	0.090	0.01 (0.01)	0.626	0.00 (0.02)	0.905	0.03 (0.03)	0.397
BMI (kg/m2)	**0.55 (0.03)**	**<0.001**	**0.56 (0.04)**	**<0.001**	**0.52 (0.10)**	**<0.001**	**0.59 (0.05)**	**<0.001**	**0.611 (0.05)**	**<0.001**	**0.52 (0.10)**	**<0.001**
Missing teeth (n)	−0.02 (0.03)	0.559	0.01 (0.03)	0.853	−0.08 (0.06)	0.103	−0.03 (0.03)	0.445	−0.02 (0.04)	0.617	−0.05 (0.08)	0.480

*p* < 0.05 denoted in bold.

**Table 5:** Mediation analysis of age for the relationship of the number of missing teeth with systolic blood pressure (SBP) and diastolic blood pressure (DBP).

**Exposure: Missing Teeth/Outcome: Systolic Blood Pressure/Mediator: Age**				
**Effect**	**Estimate**	**SE**	***p*-Value**	**95% CI**
Exposure → Mediator	1.25	0.18	<0.001	1.21–1.28
Mediator → Exposure	0.36	0.12	<0.001	0.34–0.39
Total Effect	0.50	0.02	<0.001	0.45–0.55
Direct Effect	0.05	0.03	0.073	−0.00–0.10
Mediated Effect	0.45	0.02	-	0.42–0.49
AB/C (Age percentage mediated) = 80%				
**Exposure: Missing Teeth/Outcome: Diastolic Blood Pressure/Mediator: Age**				
**Effect**	**Estimate**	**SE**	***p*-Value**	**95% CI**
Exposure → Mediator	1.25	0.02	<0.001	1.21–1.28
Mediator → Exposure	0.06	0.01	<0.001	0.04–0.07
Total Effect	0.08	0.01	<0.001	0.05–0.11
Direct Effect	0.01	0.02	0.726	−0.03–0.04
Mediated Effect	0.07	0.01	-	0.05–0.09
AB/C (Age percentage mediated) = 87.5%				

β—standardized estimates; 95% CI—95% confidence interval; SE—standard error.

## Data Availability

Data available on request due to restrictions.

## Supplementary Materials

The following are available online at https://www.mdpi.com/1660-4601/18/1/285/s1, Table S1: STROBE Statement—checklist of items that should be included in reports of case–control studies.
